# Effects of training with a rehabilitation device (Rebless®) on upper limb function in patients with chronic stroke: A randomized controlled trial

**DOI:** 10.1097/MD.0000000000038753

**Published:** 2024-06-28

**Authors:** Jong Yoon Chang, Min Ho Chun, Anna Lee, Ahro Lee, Chang Min Lee

**Affiliations:** a Department of Rehabilitation Medicine, Asan Medical Center, University of Ulsan College of Medicine, Seoul, Republic of Korea; b Asan Institute for Life Sciences, Asan Medical Center, University of Ulsan College of Medicine, Seoul, Republic of Korea; c Research Institute of Future City and Society, Yonsei University, Seoul, Republic of Korea; d PlayIdeaLab Incorporation, Seoul, Republic of Korea.

**Keywords:** robotics, stroke, stroke rehabilitation, upper limb function

## Abstract

**Background:**

Upper limb dysfunction is one of the most common sequelae of stroke and robotic therapy is considered one of the promising methods for upper limb rehabilitation.

**Objective:**

This study aimed to explore the clinical effectiveness of upper limb training using a rehabilitation robotic device (Rebless®) for patients with stroke.

**Methods:**

In this prospective, unblinded, randomized controlled trial, patients were randomly assigned to receive robotic training (experimental group, n = 15) or conventional therapy (control group, n = 15). Both groups received upper limb training lasting for 30 minutes per session with a total of 10 training sessions within 4 weeks. Motor function, functional evaluation, and spasticity were clinically assessed before and after the training. Cortical activation was measured using functional near-infrared spectroscopy at the 1st and 10th training sessions.

**Results:**

The experimental group demonstrated a significant improvement in the Fugl–Meyer assessment-upper extremity score and the modified Ashworth scale grade in elbow flexors. The cortical activity of the unaffected hemisphere significantly decreased after 10 training sessions in the experimental group compared with the control group.

**Conclusions:**

The experimental group showed significant improvement in the Fugl–Meyer assessment-upper extremity score and spasticity of elbow flexors and had significantly decreased cortical activity of the unaffected hemisphere. Training with Rebless® may help patients with chronic stroke in restoring upper limb function and recovering the contralateral predominance of activation in motor function.

## 1. Introduction

Stroke is a cerebrovascular disease accompanied with circulatory problems, causing ischemia or hemorrhage. Impairment, paralysis of body parts, abnormal muscle tone, abnormal posture, decreased movement, and decreased coordination may also occur.

Among the neurological disorders presenting after stroke, impaired upper limb function is one of the most common sequelae, in which 69% of patients with stroke admitted to a rehabilitation center presented with impaired upper limb function.^[1,2]^ Upper limb function is closely correlated with activities of daily living (ADLs), as most of ADLs require upper limb function such as eating and grooming, and upper limb function loss causes problems in independent daily living and limited participation in social activities.^[3]^

Gradual resistance training and repeated training such as reaching and object manipulation are used to improve the upper limb strength of patients with stroke. Gradual resistance training can be difficult in patients with weak muscle strength. However, repeated training can be performed by assistive devices, such as robotic devices, even for patients with weakness. Repeated training is known to improve functional ability in patients with stroke.^[4]^ In addition, robotic therapy has been suggested as a promising approach for the rehabilitation of upper limb function, as it can provide complex but controlled multisensory stimulation.^[5]^ A previous study reported that robotic therapy is effective for the treatment of upper limb paresis in patients with stroke.^[6]^

Many patients with chronic stroke suffer from upper limb paresis; however, they often do not receive sufficient upper limb rehabilitation training from occupational therapists during the chronic phase due to decreased participation following the acute and subacute periods.^[7]^ Therefore, in this study, we applied robotic training for patients with chronic stroke.

Functional near-infrared spectroscopy (fNIRS) is an optimal brain monitoring technique that indirectly analyzes brain cortical activity, similar to functional magnetic resonance imaging (fMRI).^[8]^ fNIRS can gauge regional hemodynamic shifts in oxygenated hemoglobin and deoxygenated hemoglobin that are triggered by neural activity during a task through its use of infrared light, while avoiding the need for excessive constraints.^[9]^ By using fNIRS, we could analyze the cortical activity of the brain in real time while an individual is undergoing rehabilitation training with a device.^[10]^

Several studies have utilized fNIRS to compare cortical activity before and after upper limb training with robotic devices in patients with stroke.^[11–15]^ However, there is a particular paucity of research comparing the effects of robotic versus conventional devices on cortical activity during upper limb rehabilitation in patients with stroke.

This study aimed to explore the clinical effectiveness of upper limb training using Rebless®, a commercially available rehabilitation robotic device for stroke patients, by assessing cortical activity via fNIRS and functional outcomes through a randomized controlled trial comparing robotic and conventional devices.

## 2. Methods

### 2.1. Ethics statement

The study was registered with the Clinical Research Information Service database (KCT0007053). The Asan Medical Center Institutional Review Board approved this study (No. 2022-0080), and all patients provided written informed consent. The funding organizations did not influence the analysis or writing of the paper, as they did not provide any feedback or comment.

### 2.2. Participants

The study enrolled 30 patients with chronic stroke (16 women and 14 men) in the outpatient department of Asan Medical Center’s Department of Rehabilitation Medicine. Patients were eligible to participate in the study if they had experienced a hemorrhagic or ischemic stroke, >24 months after onset, had stroke-affected arm paresis, aged ≥19 years, and had spasticity on their elbow or wrist, which is equal to or lower than modified Ashworth scale (MAS) grade 3, which indicates considerable increase in muscle tone making passive movement difficult.

Patients were excluded if they had aphasia or severe cognitive impairment, which interferes with communication, serious medical conditions, neurological or musculoskeletal diseases that may affect training, or if they could not perform other upper limb training tasks. Patients participating in a medical device study with the same indication (stroke) as this study, within 30 days after the end of the visit were also excluded.

Participants could stop the intervention in the following situations: (1) the participant or his/her representative demands discontinuation, (2) the participant withdraws consent, (3) the clinical investigation is difficult due to severe adverse events or complications, (4) the disease is deteriorating and continuation of the study is difficult, (5) the inclusion and exclusion criteria were violated during the clinical investigation, (6) the participant refused to comply with the instructions of the principal investigator, and (7) follow-up is not possible during the investigation.

### 2.3. Equipment

**Figure 1.**
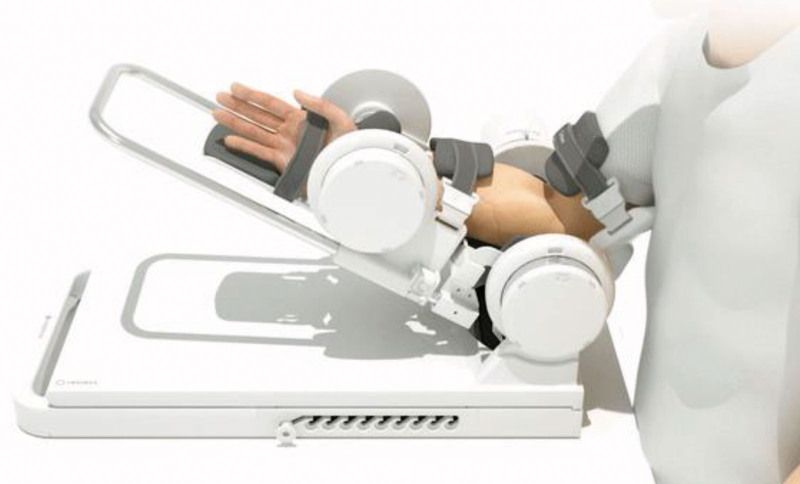
A rehabilitation robotic device, Rebless® for the experimental group.

Rebless® is a motorized type of orthopedic exercise device manufactured by H. Robotics Co., Ltd. (Fig. 1). With this device, patients can perform range of motion (ROM) and resistance exercises of the elbow and wrist joint. The device provides different operating modes for users while collecting data, such as number of repetitions, ROM, and level of resistance and assistance. For both, the maximum level of resistance and assistance is 10. By enabling patients to monitor their progress and observe personal improvements, these data could enhance motivation for the rehabilitation process and restoration of motor function.

### 2.4. Randomization and masking

**Figure 2.**
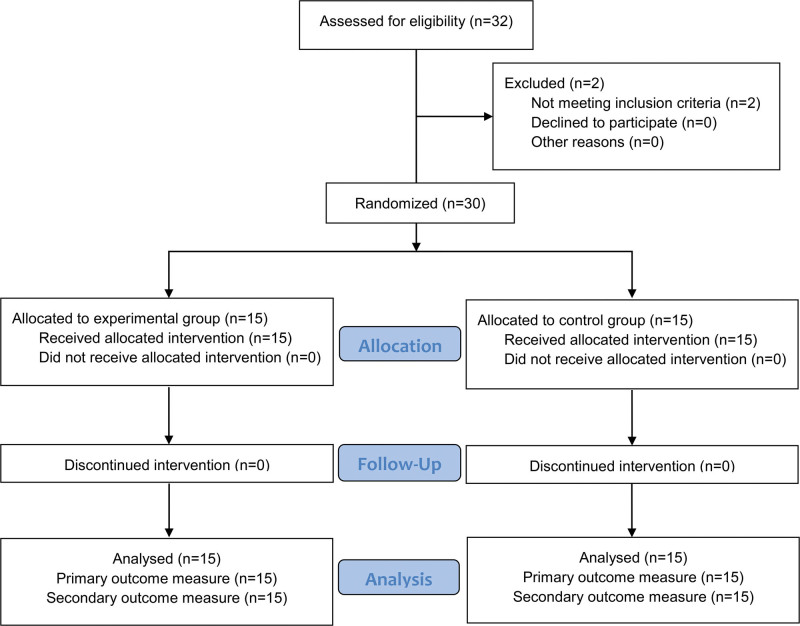
CONSORT flow diagram of the participants.

In this prospective, unblinded, randomized controlled trial, patients were allocated randomly to receive robotic training (experimental group, n = 15) or conventional therapy (control group, n = 15) (Fig. 2). The purpose of the random assignment was to compare the collected information as reliably as possible and was determined by a random check extracted by a computer. Neither patients nor clinicians evaluating the patients were blinded to the group allocation.

### 2.5. Procedure and interventions

Both groups received upper limb training lasting for 30 minutes per session, with a total of 10 training sessions, within 4 weeks. The experimental group performed rehabilitation training for 30 minutes per session with Rebless®. In the control group, the treatment of the hemiparetic arm was substituted with Motomed®, a commercially available ergometer for upper limb training.

The training setting of Rebless® was as follows: At visit 1, the experimental group started Rebless® training in the active mode, without assistance or resistance. If it was possible to train in this setting, we added one more level of resistance, until the maximum resistance that the patient could tolerate. If it was impossible to train in the setting without assistance or resistance, 2 more levels of assistance were added until the patient could perform the training. From visits 2 to 9, one more level of resistance was added or 2 levels of assistance were removed from the setting of the previous visit. The patients received training in elbow flexion, elbow extension, wrist flexion and wrist extension for 7 minutes 30 seconds each, for a total of 30 minutes. Moreover, 30 seconds were spent in measuring the ROM and setting the level of resistance or assistance, and patients received training for 7 minutes.

### 2.6. Measurements

Baseline characteristics—age, sex, height, weight, body mass index, time poststroke, stroke etiology (ischemia and hemorrhage), and affected side—were collected. Training duration, ROM, level of assistance and resistance, and number of repetitions were recorded at every visit.

Motor function, functional evaluation and spasticity were clinically assessed before initial training and after final training for the primary outcome measures by a clinician. Motor function was assessed by the Fugl–Meyer assessment of the upper extremity (FMA-UE), which is a performance-based index designed for evaluating impairments in patients with stroke, and it is scored on a scale of 0 to 66.^[16]^ The assessment is intended to evaluate balance, sensation, motor functioning, and joint functioning of patients who have hemiplegia after experiencing a stroke.

The modified Barthel index (MBI) was used to evaluate ADL function, and it is a reliable scale that ranges from 0 to 100. A higher MBI score represents greater independence in performing ADLs.^[17]^ The spasticity of elbow flexors, elbow extensors, wrist flexors, and wrist extensors of the affected upper extremity was measured by MAS (grading 0, 1, 1+, 2, 3, and 4), with higher grades indicating greater spasticity.^[18]^

The ROM of the elbow flexion, elbow extension, wrist flexion, and wrist extension of the affected arm were measured using a Trigo goniometer adapter. The motricity index for upper extremities (MI_Upper), a scale ranging from 0 to 99, was used to evaluate the function of the affected upper limb.^[19]^

For the secondary outcome measure, fNIRS was used to measure the cortical activation during the 1st and 10th training sessions (2 times). All participants filled out a user satisfaction questionnaire at the end of the training. Throughout the study, patients’ safety and any adverse effects associated with the rehabilitation treatment were monitored.

### 2.7. Data acquisition with fNIRS

**Figure 3.**
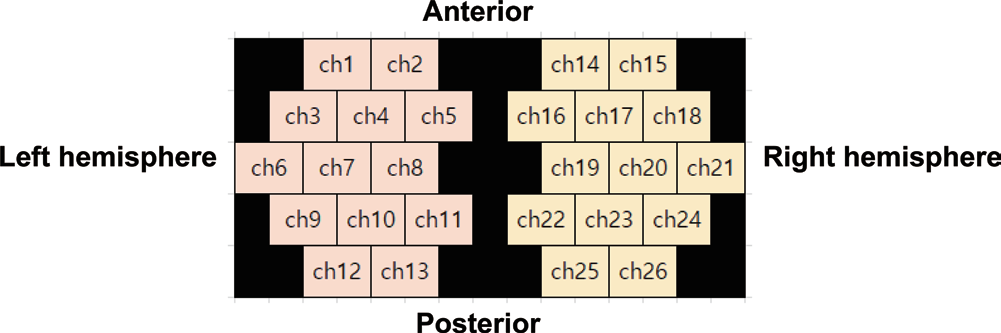
Topographical layout of the 32-optode placement covering the motor cortex, resulting in 26 channels.

In this study, a continuous-wave fNIRS system (NIRScout, NIRx Medical Technologies LLC, Germany) was used. Infrared light wavelengths were 830, 808, 780, and 685 nm as in a previous study.^[20]^ To minimize the effect of surrounding light and accurately position the optode based on the international 10 to 20 system, a cap made of black elastic material was used.^[21]^ The 26 channels with a total of 26-optode templates were arranged bilaterally over the frontal and parietal areas. Each optode was 2.0 cm apart (Fig. 3).

### 2.8. Data processing and block average of fNIRS

**Figure 4.**
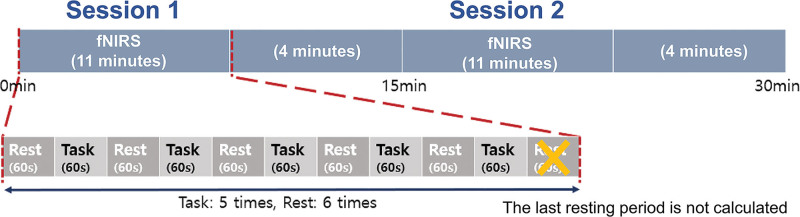
fNIRS (Functional near-infrared spectroscopy) measurement was performed while the patients executed the 11 cycling tasks. Each period started with a rest period (30 seconds) and a training period (30 seconds) and repeated in 2 sessions. The last rest period (30 seconds) was not calculated.

The block paradigm design was used. The experiment consisted of 2 sessions of 5 cycles, each consisting of rest (60 seconds)–task (60 seconds) (Fig. 4). A single measurement lasted 600 seconds. fNIRS data were analyzed by MATLAB. Data were extracted from 5 segments of rest and 5 segments of task and averaged by the block averaged method. These averaged data from 2 sessions were also averaged.

To obtain the most meaningful signal, the quality of data collected for each channel during the task was evaluated in this process. Signal errors were removed from the data, such as spikes or discontinuities caused by environmental influences, to maximize reliability. Additionally, a bandpass filter was used to remove slow drift and noise. The modified Beer–Lambert law was applied to calculate the amount of oxygen change.^[22]^ With this equation, the change in the detected values for each wavelength was used to calculate concentration changes. Through signal processing, the electrical signal that was observed was converted into a value that reflects the changes in oxygen levels. We assumed that the measurement began at 0 seconds, as it is a relative value.

### 2.9. Statistical analysis

The sample size was calculated as previously described.^[3,23,24]^ A study with the minimum number of participants within the limit of satisfying the purpose of the study by referring to the previous research results is desirable. Considering the dropout rate of approximately 20% during the clinical study period, a total of 30 participants, 15 per group, were planned to be enrolled.

IBM SPSS Statistics for Windows version 25 (IBM Corp., Armonk, NY) was used to analyze data. For baseline characteristics, the Mann–Whitney *U* test and the chi-square test were used, as appropriate, to compare the experimental group and the control group.

For the primary outcomes, a Wilcoxon signed-rank test was used to assess the differences between outcome measurements at baseline and after training. A Mann–Whitney *U* test was used to evaluate the post-training differences between the experimental group and the control group. For the fNIRS data, a two-way analysis of variance was used to measure the difference between pre- and post-training cortical activity levels of the experimental group and the control group. Statistical significance was defined as a *P*-value of <.05.

## 3. Results

Between March 2022 and August 2022, a total of 30 patients with chronic stroke (16 women and 14 men, mean age of 66.2 ± 5.4 (mean ± SD) years) were enrolled in the study. All participated successfully completed 10 training sessions and fNIRS measurements without any adverse events or dropouts.

The baseline characteristics of the experimental and control groups were not significantly different, including age, sex, height, weight, body mass index, types of stroke, affected side, and time poststroke. The mean age values of the experimental and control groups were 65.2 ± 5.4 and 67.2 ± 5.4 years, respectively. Both groups included 7 men and 8 women. The average time poststroke was 186.3 ± 94.5 months in the experimental group and 192.1 ± 49.0 months in the control group. The clinical and demographic characteristics of both groups are shown in Table 1.

**Table 1:** Patients’ baseline characteristics.

	Experimental group (n = 15)	Control group (n = 15)	*P*-value
Age (years)	65.2 ± 5.4	67.2 ± 5.4	.37
Sex (men: women)	7:8	7:8	1*
Height (cm)	160.9 ± 8.4	158.7 ± 8.7	.42
Weight (kg)	63.9 ± 10.9	62.6 ± 11.9	.71
BMI† (kg/m²)	24.5 ± 3.4	24.7 ± 2.8	.65
Infarction (%)	53.3	53.3	1*
Hemorrhage (%)	46.7	46.7	1*
Hemiparetic side (right: left)	8:7	10:5	.46*
Time poststroke (months)	186.3 ± 94.5	192.1 ± 49.0	.72

Values are presented as mean ± standard deviation.

* Chi-square test; otherwise Mann–Whitney *U* test.

† Body mass index (BMI) is a person’s weight in kilograms divided by the square of height in meters.

### 3.1. Primary outcomes: Functional outcomes

All baseline assessments of the experimental and control groups were not significantly different. The baseline and posttreatment results of the clinical measurements are shown in Table 2. Comparing pre- and post-rehabilitation treatment, the FMA-UE, MBI, MI_Upper, MAS grade in the elbow extensors and wrist extensors, and ROM of elbow flexion and wrist extension in the experimental group significantly improved. However, except for the FMA-UE, the improvement was not significant when compared with the control group. In the control group, none of the assessments showed a significant change after the training.

**Table 2:** Outcome measures at baseline and post-training.

	Experimental group (n = 15)			Control group (n = 15)			*P*-value
	Baseline	Post	*P*-value	Baseline	Post	*P*-value	
FMA-UE	27.67 ± 8.41	32.00 ± 9.23	<.001*	22.07 ± 10.12	23.76 ± 9.47	.708	.003†
MBI	88.67 ± 6.07	90.27 ± 4.40	.031*	90.60 ± 4.64	90.87 ± 4.73	.832	.093
MI_Upper	62.40 ± 12.11	68.07 ± 11.36	.002*	61.00 ± 11.58	64.33 ± 10.86	.413	.164
MAS (elbow flexor)	0.57 ± 0.56	0.27 ± 0.46	.063	0.57 ± 0.56	0.77 ± 0.50	.321	.005†
MAS (elbow extensor)	1.10 ± 0.66	0.60 ± 0.60	.004*	1.37 ± 0.35	1.20 ± 0.46	.353	.053
MAS (wrist flexor)	0.63 ± 0.55	0.47 ± 0.52	.250	0.87 ± 0.88	0.77 ± 0.70	.844	.609
MAS (wrist extensor)	1.23 ± 0.62	0.77 ± 0.59	.002*	1.40 ± 0.89	1.23 ± 0.65	.745	.164
ROM (elbow flexor)	128.33 ± 12.63	134.00 ± 9.49	.004*	123.33 ± 14.72	126.00 ± 14.29	.436	.241
ROM (elbow extensor)	0.67 ± 1.76	1.00 ± 2.07	1	0.00 ± 0.00	0.00 ± 0.00	1	.317
ROM (wrist flexor)	54.33 ± 19.72	58.00 ± 20.16	.125	47.33 ± 20.52	49.67 ± 19.86	.786	.980
ROM (wrist extensor)	37.33 ± 22.59	45.67 ± 16.35	.008*	31.00 ± 17.65	33.67 ± 16.42	.558	.119

Values are presented as mean ± standard deviation.

FMA-UE = Fugl–Myer Assessment-Upper Extremity, MAS = modified Ashworth scale, MBI = modified Barthel index, MI_Upper = motricity index for the upper extremities, ROM = range of motion.

* *P* < .05, by the Wilcoxon signed-rank test, for baseline versus post-training.

† *P* < .05, by the Mann–Whitney *U* test, for the difference (post-training) of the experimental group versus control group.

The experimental group demonstrated a significant increase in the FMA-UE score compared with the control group, especially in wrist and coordination/speed assessment (*P*-value = .003).

The MAS grade in elbow flexors decreased more in the experimental group, which shows improvement in elbow flexor spasticity compared with the control group (*P*-value = .005). The change value in the MBI, MI_Upper, spasticity of elbow extensors, wrist flexors, and wrist extensors, and ROM of elbow flexion, elbow extension, wrist flexion, and wrist extension pre- and post-rehabilitation treatment did not show significant difference between the 2 groups.

### 3.2. Secondary outcomes: fNIRS data

**Figure 5.**
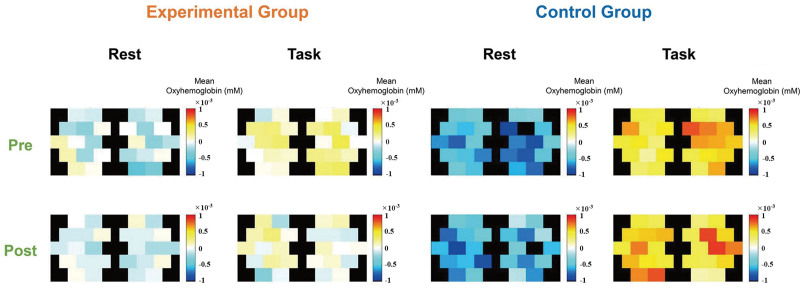
Averaged cortical activity measured by functional near-infrared spectroscopy in the experimental and control groups during a rest and a task period, before (pre) and after (post) 10 training sessions.

**Figure 6.**
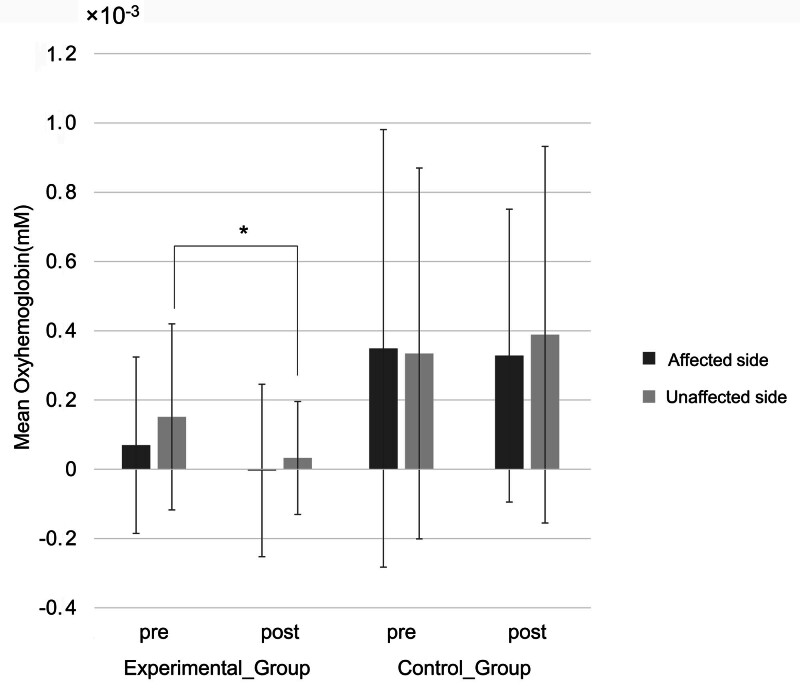
Changes in the cortical activity measured by functional near-infrared spectroscopy in the affected and unaffected hemispheres. The mean oxyhemoglobin concentration of the unaffected hemisphere measured by the functional near-infrared spectroscopy significantly decreased after the training in the experimental group compared with the control group (*P-value = .04, by the two-way analysis of variance).

The cortical activity measured by fNIRS was higher in the control group than in the experimental group regardless of the resting or tasking period (Fig. 5). We assume that this is due to the movement of the participants during training, which was much bigger in the control group. Nevertheless, the cortical activity of the unaffected hemisphere measured by the fNIRS significantly decreased after the training in the experimental group, which was not observed in the control group (Fig. 6).

## 4. Discussion

This study compared changes in functional evaluations and cortical activation measured by fNIRS after training with a robotic device or a conventional device. Several studies have utilized fNIRS to compare cortical activity before and after upper limb training with robotic devices in patients who have experienced a stroke.^[11–15]^ One study conducted robot-assisted therapy in stroke patients with hemiplegia, using fNIRS to compare cortical activity between groups with severe and moderate impairments.^[13]^ However, to the best of our knowledge, few studies are using fNIRS to compare cortical activity after training with a robotic device and conventional device for upper limb rehabilitation in patients with stroke. This study showed that training the upper extremity with Rebless® is more effective in improving FMA-UE and spasticity of elbow flexors than conventional therapy. In addition, the cortical activity of the unaffected hemisphere was significantly decreased after training with Rebless®.

The MBI, MI_Upper, and ROM of the elbow and wrist did not show any improvement in both groups. This is probably because the participants all had chronic stroke, and the average time poststroke was 186.3 ± 94.5 months in the experimental group and 192.1 ± 49.0 months in the control group. Most of the improvement of function in patients with stroke is observed within 6 months. A previous study showed that the stroke subtype-, sex-, and age-adjusted Barthel index curve increased sharply until weeks 8 to 24 and subsequently plateaued.^[25]^ Therefore, making some improvements in function for patients with chronic stroke compared with those with acute or subacute stroke would be difficult.

As participants could perform wrist exercise by Rebless®, which was not possible with a conventional upper limb ergometer in the control group, the FMA-UE score and wrist and coordination/speed assessment of FMA-UE showed significant improvement in the experimental group. A previous study compared robotic hand exoskeleton to conventional therapy and showed significant increase in wrist and hand domain of FMA-UE, because intensive and repetitive training of the wrist was possible with the robotic device.^[26]^ Even though this improvement of FMA-UE did not lead to a difference in the MBI score in the present study, the fine motor function would have some improvement in the experimental group, even though no evaluation was conducted.

Nevertheless, the ROM of the elbow and wrist joints did not change in both groups, and the decrease in the spasticity of elbow flexors in the experimental group was thought to be due to more active ROM exercise conducted by Rebless®. When training with Rebless®, we checked the ROM of the elbow and wrist joints before the training and repeated ROM as possible. However, there was no such process in the control group. A previous study showed that flexibility exercises can relieve muscle spasticity problems in patients with stroke.^[27]^

Cortical excitability was found to correlate with the potential for functional recovery in patients with chronic stroke.^[28]^ In our study, the movement of the participants in the control group was much bigger than the movement in the experimental group because of the characteristics of the devices. A conventional upper limb ergometer involves more proximal joints such as the shoulder and elbow, whereas Rebless® does not involve the shoulders but involves distal joints—elbow and wrist. Therefore, the cortical activation of both resting and tasking state were higher in the control group than in the experimental group. Nevertheless, the cortical activity of the unaffected hemisphere measured by the fNIRS significantly decreased after the training in the experimental group. A study showed that in patients with stroke, the cortical activity of the unaffected hemisphere was also activated as much as the affected hemisphere while moving the affected hand.^[29]^

Another study of upper limb training, which acquired neurophysiological measures of cortical excitability by motor evoked potential (MEP) using transcranial magnetic stimulation and resting motor threshold pre- and post-therapy, showed that the MEP amplitude of the contralesional hemisphere decreased considerably in both groups, even though it was not significant.^[26]^ This study presented that a decrease in MEP amplitude in the contralesional hemisphere after the training might indicate a decrease in cortical excitability, which was also shown in the present study.^[30,31]^ A decrease in cortical excitability of the unaffected hemisphere may suggest the recovery of the contralateral predominance of the activation in motor function.

This study has several limitations. First, the participants were patients with chronic stroke so that neuroplasticity was not as active as in patients with acute stroke. There might have been different results if the study was conducted with patients with acute stroke. Second, a total of 10 training sessions might not be enough to make a difference in patients with chronic stroke. Third, the movement when training with a conventional upper limb ergometer was much bigger than that when training with Rebless®, which made much more cortical activation. This made the comparison more difficult in the 2 groups. In addition, neither patients nor clinician evaluating them were blinded, potentially impacting the assessment. Despite these limitations, training with Rebless® has the potential in improving wrist and hand function, spasticity of elbow flexors, and recovery of the contralaterality of motor function.

## 5. Conclusion

After training with Rebless®, a rehabilitation robotic device for the upper limb, the FMA-UE score and spasticity of elbow flexors showed significant improvement compared with training using a conventional device. Furthermore, the cortical activity of the unaffected hemisphere was significantly decreased in the experimental group. Training with Rebless® may help patients with chronic stroke in restoring upper limb function and recovering the contralateral predominance of the activation in motor function. The sustained rehabilitation training for patients with chronic stroke, especially using rehabilitation robot, is recommended because it improves upper limb function in patients with chronic stroke.

## Author contributions

**Data curation:** Anna Lee, Ahro Lee, Chang Min Lee.

**Formal analysis:** Chang Min Lee.

**Funding acquisition:** Min Ho Chun.

**Investigation:** Chang Min Lee.

**Methodology:** Min Ho Chun.

**Project administration:** Min Ho Chun.

**Resources:** Anna Lee, Ahro Lee.

**Supervision:** Jong Yoon Chang, Min Ho Chun.

**Writing – original draft:** Jong Yoon Chang.

**Writing – review & editing:** Min Ho Chun.
